# Supplementary figures and images for: Closed loop motor-sensory dynamics in human vision
Source: PLoS One. 2020 Oct 15;15(10):e0240660. doi: 10.1371/journal.pone.0240660 (PMC7561174; doi:10.1371/journal.pone.0240660)

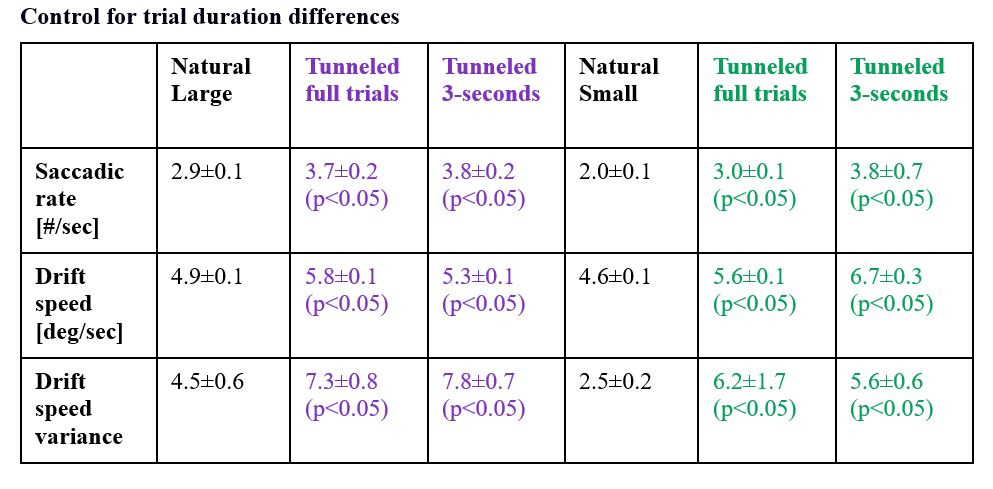

Supplement: S1 Table — Related to Fig 4. The analyses described in Fig 3 were repeated for the first 3 s of the tunneled conditions, a time period equal to the duration of natural viewing trials. P values represent the probability that the values measured in the relevant tunneled condition were drawn from the same distribution as those measured in the natural viewing conditions (two tailed t-tests for means and two tailed f-tests for variances). (JPG) [file pone.0240660.s003.JPG]

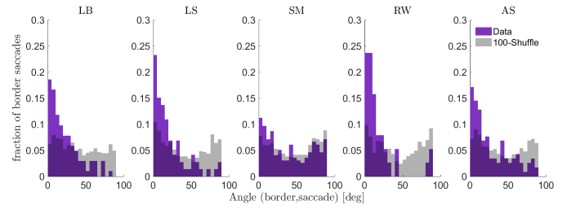

Supplement: S1 Fig — Related to Fig 1B and 1C. Distributions of the angles between the orientation of the border scanned during a pause and the direction of the immediately following saccade [data shown in purple, shuffled data (saccade directions were shuffled before angle computation; average of 100 repetitions is depicted) in gray]. Data for each is presented. All distributions are statistically different, p<0.05, two-sample Kolmogorov-Smirnov tests. (JPG) [file pone.0240660.s004.jpg]

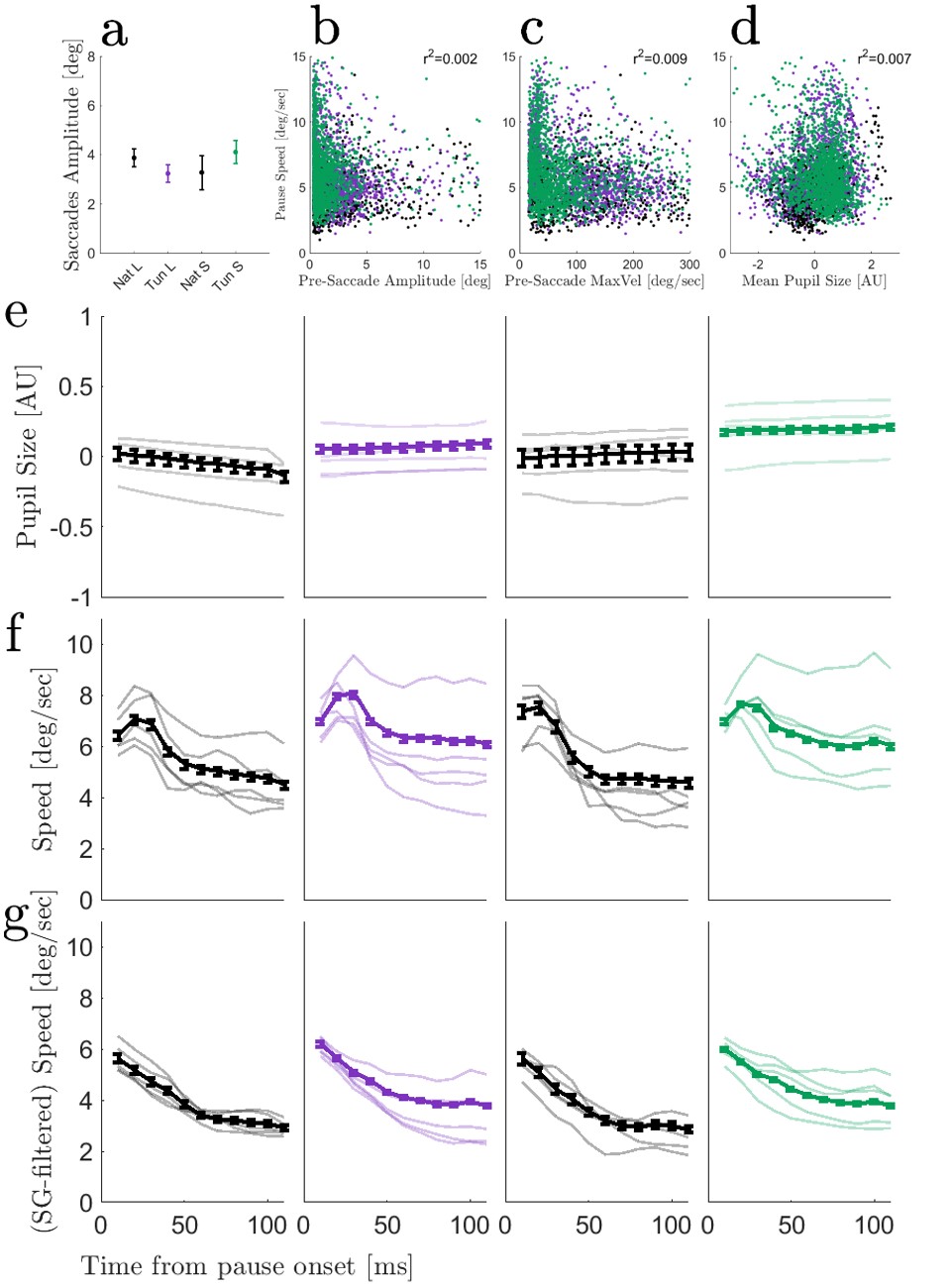

Supplement: S2 Fig — Related to Fig 3. (a) The mean amplitude of the preceding saccades of all pauses in each of the four experimental conditions; no significant difference was found (p > 0.1, two-tailed t-test); similarly, no significant difference was found for the maximal saccade speed (p > 0.1, two-tailed t-test). (b-d) Each data point represents a single pause (mean pause speed versus (b) the amplitude of the preceding saccade, (c) the maximal speed of the preceding saccade (d) mean pupil size during the pause). R2 < 0.01 in all cases. Colors as in Fig 3. (e) Mean within-pause instantaneous pupil size (f) Mean within-pause instantaneous drift speed (no correlation with the mean within-pause instantaneous pupil size, R2 = 0.02, p = 0.55) (g) Lower bound of the mean within-trial instantaneous drift speed, calculated from the filtered data (a third order Savitzky-Golay filter with window size of 3 samples [45]). (JPG) [file pone.0240660.s005.jpg]

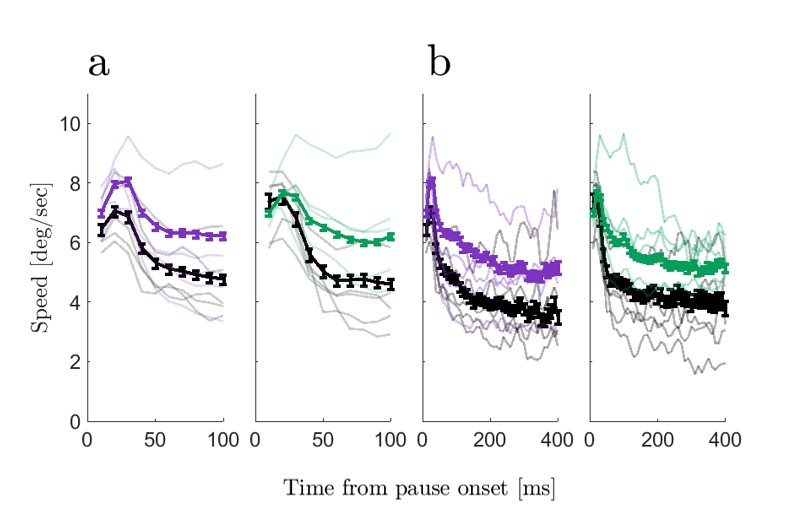

Supplement: S3 Fig — Related to Fig 3. (a) Mean within-pause instantaneous drift speeds presented for large (left) and small (right) objects, depicted for 0<t<100 ms from pause onset (colors as in Fig 3). Error-bars denote SEMs across pauses. Light colors show individual means per subject. (b) same as (a) for 0<t<400 ms. (JPG) [file pone.0240660.s006.jpg]
